# Regulation of Adipogenesis by Quinine through the ERK/S6 Pathway

**DOI:** 10.3390/ijms17040504

**Published:** 2016-04-13

**Authors:** Xiaomin Ning, Jingjing He, Xin’e Shi, Gongshe Yang

**Affiliations:** Laboratory of Animal Fat Deposition and Muscle Development, College of Animal Science and Technology, Northwest Agriculture and Forestry University, Yangling 712100, China; xmning@hotmail.com (X.N.); wsjj1987@hotmail.com (J.H.); xineshi@163.com (X.S.)

**Keywords:** quinine, adipogenesis, T2R106, ERK/S6 signaling

## Abstract

Quinine is a bitter tasting compound that is involved in the regulation of body weight as demonstrated in *in vivo* animal models and *in vitro* models of the adipogenic system. Arguments exist over the positive or negative roles of quinine in both *in vivo* animal models and *in vitro* cell models, which motivates us to further investigate the functions of quinine in the *in vitro* adipogenic system. To clarify the regulatory functions of quinine in adipogenesis, mouse primary preadipocytes were induced for differentiation with quinine supplementation. The results showed that quinine enhanced adipogenesis in a dose dependent manner without affecting lipolysis. The pro-adipogenic effect of quinine was specific, as other bitter tasting agonists had no effect on adipogenesis. Moreover, the pro-adipogenic effect of quinine was mediated by activation of ERK/S6 (extracellular-signal-regulated kinase/Ribosomal protein S6) signaling. Knockdown of bitter taste receptor T2R106 (taste receptor, type 2, member 106) impaired the pro-adipogenic effect of quinine and suppressed the activation of ERK/S6 signaling. Taken together, quinine stimulates adipogenesis through ERK/S6 signaling, which at least partly functions via T2R106.

## 1. Introduction

Obesity has become one of the biggest global challenges affecting human health in recent decades. Obesity increases the risk for various diseases, such as type 2 diabetes, atherosclerosis, hypertension, hyperlipidemia and certain types of cancers [1,2]. Society is under great economic pressure to treat obesity. Hence, there is an urgent need for effective therapeutic approaches to combat obesity and related diseases. Expansion of adipose tissue is the primary factor that contributes to the development of obesity, which is caused by adipocyte hypertrophy and hyperplasia [3,4,5]. As the main composition of adipose tissue, adipocytes are derived from preadipocytes through adipogenesis. Although regulation of adipogenesis has been extensively studied, effective therapeutic approaches are still in demand, thus further investigation of the mechanism of adipogenesis will facilitate our understanding and potential therapeutic treatment of obesity and associated metabolic syndrome.

As an active antimalarial compound, quinine is a natural alkaloid extracted from the bark of the cinchona tree [6]. Quinine is used for treatments of nocturnal leg cramps [7], and quinine is also used as a bitter compound in many taste tests [8,9,10,11]. The first study that linked quinine with metabolism was reported in 1973, they found quinine adulteration of the diet resulted in decreased body weight in LH-lesioned and intact male rats [12], thereafter, several other studies also reported that both food intake and body weight were remarkably decreased in rats consuming a quinine-supplemented diet [13,14,15,16,17]. In addition, quinine was reported to control body weight without affecting food intake in male C57BL6/J mice [18]. However, another study revealed that quinine promoted body weight gain and development of type 2 diabetes in humans, in which small doses of oral quinine sulfate led to excessive weight gain and type 2 diabetes with long-term use [19]. These data suggest that quinine may play important roles in adipogenesis and the development of obesity. However, the biological effects and mechanisms of quinine on adipogenesis have not been well studied.

The controversial effects of quinine on body weight gain and food intake in *in vivo* animal models and humans motivated us to further investigate the influences of quinine on adipogenesis in an *in vitro* model. In this study, we provide the first evidence that quinine is a positive regulator of adipogenesis in mouse primary preadipocytes functioning through ERK/S6 (extracellular-signal-regulated kinase/Ribosomal protein S6) pathway without affecting lipolysis, and uncover a possible role for the bitter receptor T2R106 (taste receptor, type 2, member 106) in mediating the effect of quinine on adipogenesis. Taken together, our data demonstrate the positive role of quinine in adipogenesis in mouse primary preadipocytes, and provide evidence of the presence of bitter taste receptors in the adipogenic system.

## 2. Results

### 2.1. Quinine Increases Differentiation of Mouse Primary Preadipocytes

**Figure 1:**
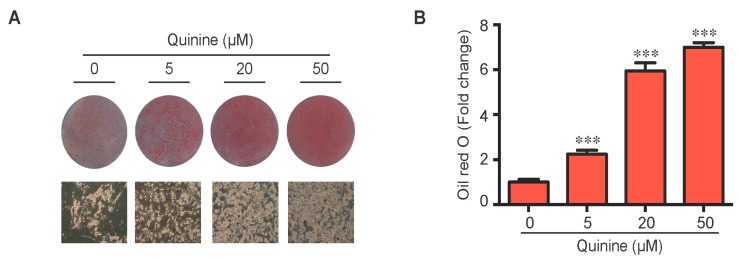

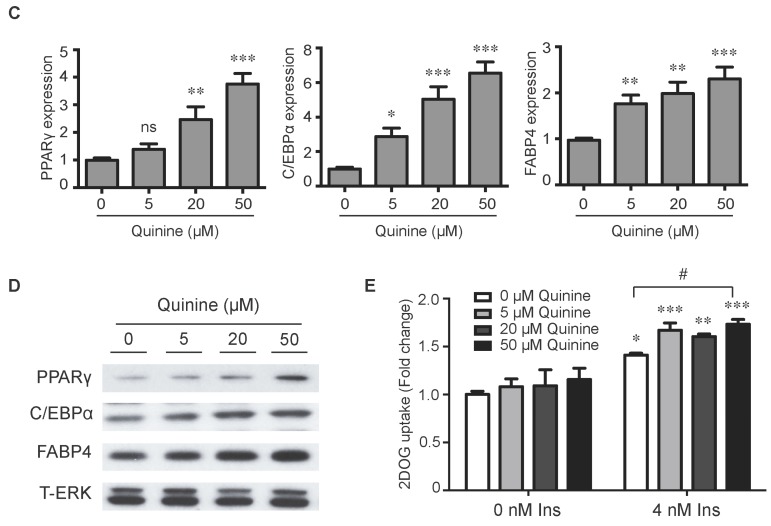
Quinine stimulated adipogenesis of mouse primary preadipocytes in a dose dependent manner. The effect of quinine on differentiation of mouse primary preadipocytes was assessed using Oil Red O stain, qRT-PCR, Western blot and glucose uptake assay, all these measurements were undertaken on Day 14 of differentiation with induction of MDI in the presence of quinine with indicated concentrations. (**A**) Oil Red O scan and micrographs (100×) showed lipid accumulation (pictures shown here represent three independent experiments); (**B**) Quantification of Oil Red O stain from three independent experiments. Data are expressed as fold change compared with 0 µM quinine (mean ± SEM; one way ANOVA, *** *p* < 0.001); (**C**) mRNA expression of adipogenic markers (PPARγ, C/EBPα and FABP4) were measured with qRT-PCR. Results are expressed as fold change compared with 0 µM quinine (mean ± SEM; *n* = 6; one way ANOVA, * *p* < 0.05; ** *p* < 0.01; *** *p* < 0.001); (**D**) The corresponding protein levels of PPARγ, C/EBPα and FABP4 were detected with Western blot. Total ERK (T-ERK) as loading control; and (**E**) Basal and insulin-stimulated 2-deoxyglucose uptake was measured as outlined in Materials and Methods. Ins represents Insulin. Results are expressed as fold change compared with cells treated with 0 µM quinine (mean ± SEM; *n* = 6; two way ANOVA; * *p* < 0.05, ** *p* < 0.01, *** *p* < 0.001 indicated 4 nM insulin compared with 0 nM insulin under each quinine concentration; # *p* < 0.05, indicated fold change compared with cells treated with 0 µM quinine under each insulin concentration).

To evaluate the role of quinine in adipocyte differentiation, mouse primary preadipocytes were induced to differentiate by an adipogenic cocktail with or without quinine for 14 days. As shown in Figure 1A,B, the addition of quinine stimulated lipid accumulation of mouse primary preadipocytes in a dose dependent manner, suggesting a positive role for quinine in adipogenesis. Additional features of differentiation were assessed by qRT-PCR and Western blot. Consistent with the morphology change, the mRNA expression levels of adipogenic markers including PPARγ (peroxisome proliferator-activated receptor γ), C/EBPα (CAAT/enhancer binding protein α) and FABP4 (fatty-acid-binding protein 4) were significantly increased (Figure 1C). Analysis of protein expression confirmed the pro-adipogenic effects of quinine in mouse primary preadipocytes (Figure 1D). We further investigated the effects of quinine on adipocyte function by characterization of glucose uptake. As shown in Figure 1E, basal glucose uptake was unaffected but insulin-stimulated glucose uptake was significantly increased by different concentration of quinine, and the highest concentration (50 µM quinine) we used had the best 2-DOG glucose uptake, which suggests quinine also enhanced adipocyte function in a dose dependent manner (Figure 1E). All these data demonstrate that quinine enhances adipogenesis both at the morphological and genetic levels, and in a dose dependent manner.

### 2.2. The Pro-Adipogenic Effects of Quinine Are Specific

**Figure 2:**
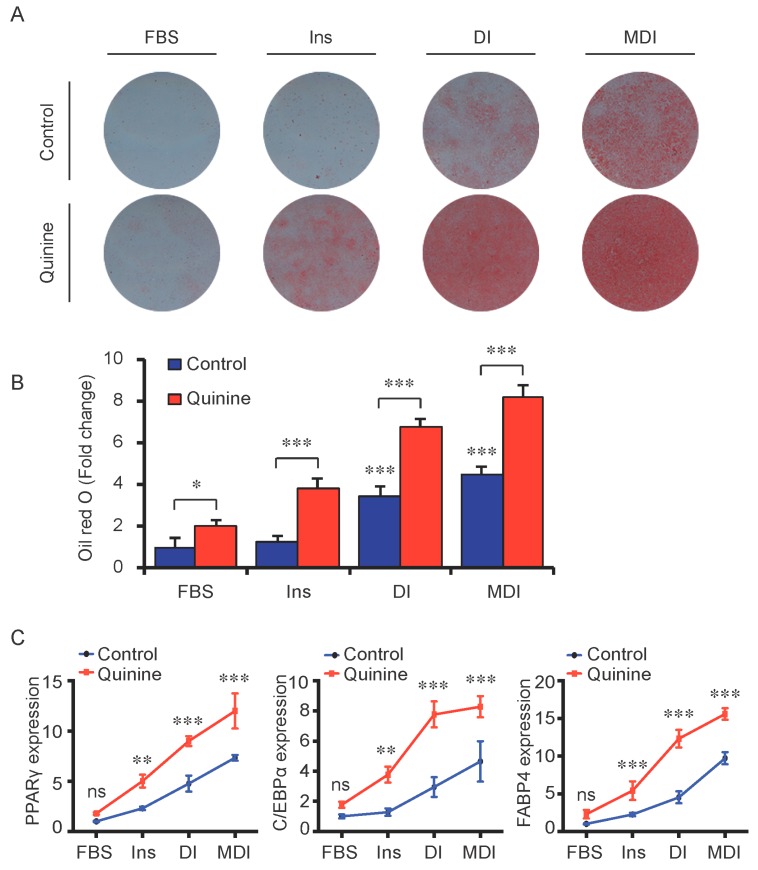
Quinine induced adipogenesis was independent of induction conditions. The effects of quinine on differentiation of mouse primary preadipocytes were assessed using Oil Red O stain and qRT-PCR on Day 14 of differentiation with different induction conditions (FBS alone, FBS + Insulin, FBS + DI and FBS + MDI) in the presence of 20 µM quinine. FBS alone was used as control. Ins represents Insulin. (**A**) Oil Red O stain showed lipid accumulation (pictures shown here represent three independent experiments). The plates were scanned after Oil Red O stain; (**B**) Quantification of Oil Red O stain from three independent experiments (mean ± SEM; one way ANOVA, * *p* < 0.05, *** *p* < 0.001); and (**C**) Total RNA was isolated and used for detection of PPARγ, C/EBPα and FABP4 mRNA expression levels with qRT-PCR (mean ± SEM; *n* = 6; two way ANOVA; ** *p* < 0.01, *** *p* < 0.001, indicated fold change compared with cells without quinine treatment under each induction condition).

**Figure 3:**
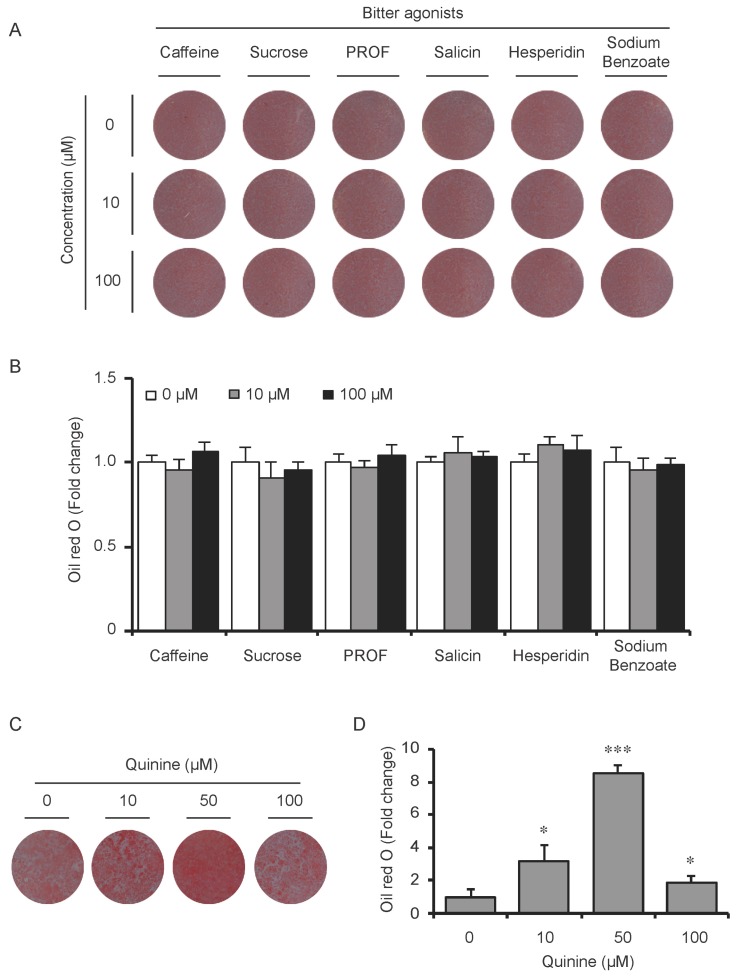
The pro-adipogenic effect of quinine was specific among bitter compounds. (**A**) Mouse 3T3-L1 preadipocytes were induced for differentiation with supplementation of different concentrations (0, 10 and 100 µM) of six different bitter agonists, caffeine, sucrose octaacetate, PROP, salicin, hesperidin and sodium benzoate. Oil Red O stain was performed to check the lipid accumulation at Day 8 of differentiation with addition of different bitter compounds. The plates were scanned after Oil Red O stain; (**B**) Quantification of Oil Red O stain from three independent experiments. Data are expressed as fold change compared with 0 µM of each bitter compound (mean ± SEM; one way ANOVA); (**C**) Oil Red O scan showed lipid accumulation (pictures shown here represent three independent experiments); and (**D**) Quantification of Oil Red O stain from three independent experiments. Data are expressed as fold change compared with 0 µM quinine (mean ± SEM; one way ANOVA, * *p* < 0.05, *** *p* < 0.001).

To test whether the pro-adipogenic effects of quinine are specific, we examined the differentiation status of mouse primary preadipocytes under diverse inductions including fetal bovine serum (FBS) alone, FBS + Insulin, FBS + DI and FBS + MDI added with 20 µM quinine. Oil Red O stain was used as indication of lipid accumulation, as shown in Figure 2A,B, stronger adipogenic inducer obviously enhanced the lipid accumulation of mouse primary preadipocytes, and with the addition of quinine, more remarkable increase of lipid accumulation was found under all induction conditions. Moreover, consistent with the morphology change, mRNA expression of adipogenic markers PPARγ, C/EBPα and FABP4 were elevated with addition of quinine in all induction media (Figure 2C). In addition, quinine is also referred to as a bitter compound [8,9,10,11], thus we further tested whether the pro-adipogenic effects of quinine are specific in comparison with other bitter compounds. To test this, several bitter compounds including caffeine, sucrose, PROF (6-propyl-2-thiouracil), salicin, hesperidin and sodium benzoate were added to culture medium with different concentrations during mouse 3T3-L1 preadipocyte differentiation. As shown in Figure 3A,B, no obvious changes were observed in cells with supplementation of caffeine, sucrose, PROF, salicin, hesperidin and sodium benzoate by Oil Red O stain under tested concentrations, even when we use diverse concentrations of each compound. Meanwhile, quinine supplementation induced adipogenesis of 3T3-L1 (Figure 3C,D), which further confirmed quinine mediated adipogenesis in mouse primary preadipocytes. These results indicate that the pro-adipogenesis effects are quinine specific.

### 2.3. Quinine Has No Effect on Lipolysis

**Figure 4:**
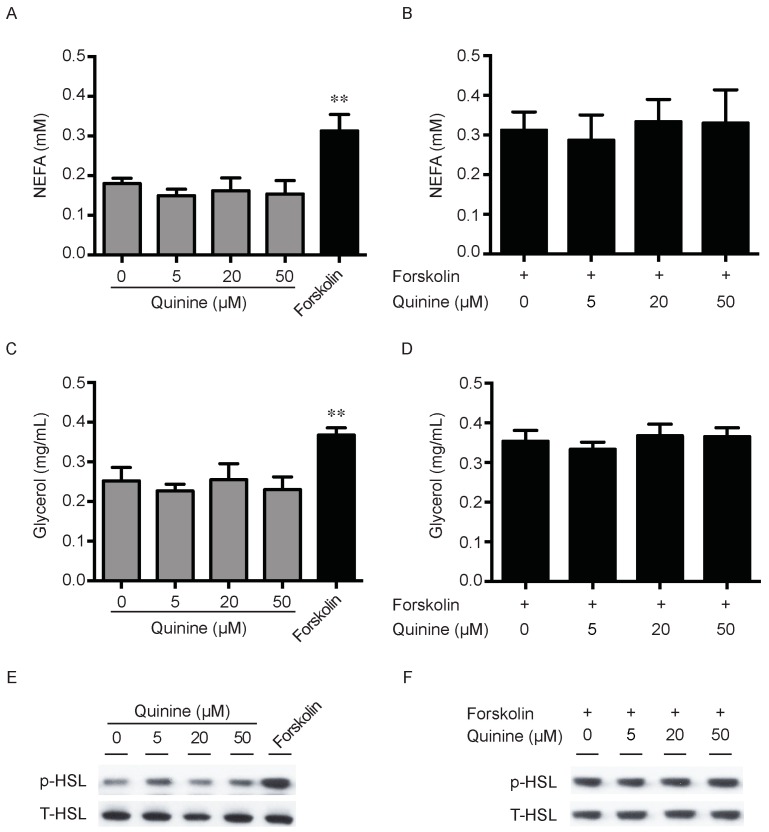
Quinine has no effect on Lipolysis. Mouse primary preadipocytes were induced for differentiation with MDI for 14 days. Mature adipocytes were serum starved for 2 h with HBSS, and then treated with the indicated concentration of quinine or 10 µM forskolin for 2 h (**A**,**C**); or treated with the indicated concentration of quinine in the presence of 10 forskolin for 2 h (**B**,**D**); cultured media were collected for measurement of NEFA or glycerol (**A**–**D**); and adipocytes were collected and lysed for Western blot assessment (**E**,**F**). (**A**) NEFA content from media was measured after quinine or forskolin treatment; (**B**) NEFA concentration was detected after adding the indicated concentration of quinine in the presence of forskolin; (**C**) glycerol content of the culture medium was assayed after quinine or forskolin treatment; and (**D**) glycerol content in the media was detected after adding the indicated concentration of quinine in the presence of forskolin. Results are expressed as fold change compared with 0 µM quinine (mean ± SEM; *n* = 6; one way ANOVA; ** *p* < 0.01) (**A**–**D**). (**E**) Phosphorylated HSL (p-HSL) was detected with Western blot after quinine or forskolin treatment. Total HSL (T-HSL) was used as control; and (**F**) p-HSL was detected by Western blot after adding the indicated concentration of quinine in the presence of forskolin.

Lipid accumulation is a chronic imbalance between triglyceride synthesis and lipolysis. There is also the possibility that the pro-adipogenic activity of quinine is due to decrease of adipocyte lipolysis. To explore this, we next measured the effects of quinine on lipolysis in fully differentiated mouse primary adipocytes. As shown in Figure 4, secretion of NEFA (non-esterifed fatty acid) in the media had no obvious changes after quinine supplementation in basal and stimulated conditions (Figure 4A,B). Consistently, glycerol content in the media showed a similar trend after quinine supplementation (Figure 4C,D). Moreover, as a lipolysis marker, phosphorylation of hormone-sensitive lipase (HSL) showed no obvious change with different concentrations of quinine treatment both in basal and stimulated conditions (Figure 4E,F). Together, these results suggest that quinine-mediated adipogenesis is independent of lipolysis.

### 2.4. Quinine Activates the ERK/S6 Signaling Pathway

**Figure 5:**
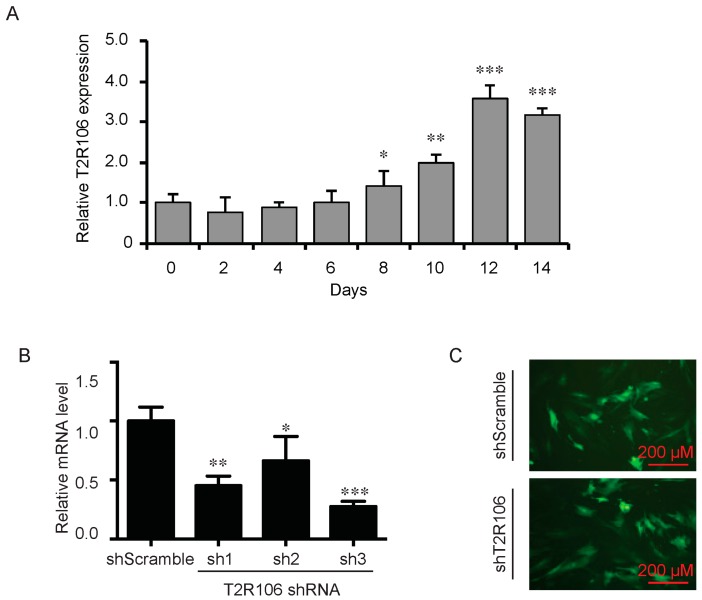
Expression and knockdown of T2R106 in mouse primary preadipocytes. (**A**) Expression of T2R106 during adipogenesis of mouse preadipocytes. Cells were induced for differentiation with MDI for 14 days, total RNA was isolated and used for detection of T2R106 mRNA expression levels with qRT-PCR (mean ± SEM; *n* = 6; two way ANOVA; * *p* < 0.05, ** *p* < 0.01, *** *p* < 0.001, indicated fold change compared with Day 0); (**B**) Knockdown efficiency of T2R106 in mouse primary preadipocytes was assessed with qRT-PCR after 48 h infection (mean ± SEM; *n* = 6; two way ANOVA; * *p* < 0.05, ** *p* < 0.01, *** *p* < 0.001, indicated fold change compared with control). Sh1, sh2 and sh3 represent T2R106 shRNA-1, shRNA-2 and shRNA-3 respectively. ShScramble represents scramble shRNA. T2R106 shRNA-3 was used for further experiments; and (**C**) Infection efficiency of T2R106 shRNA was indicated with GFP after 48 h infection.

**Figure 6:**
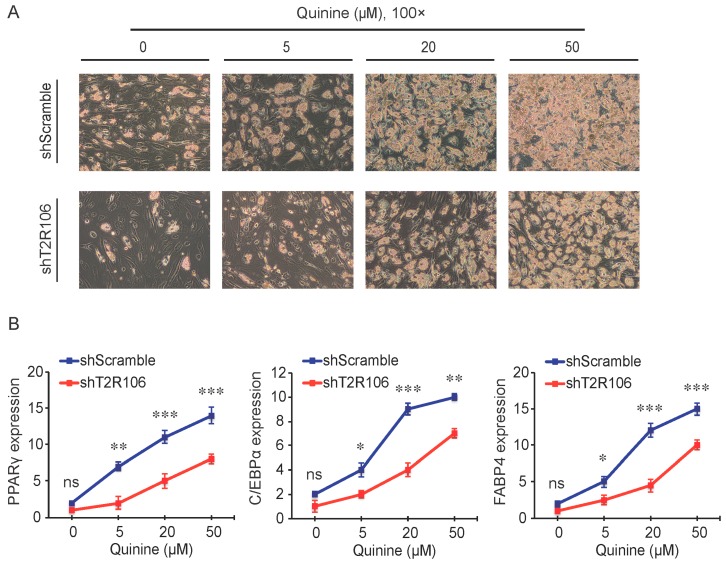
T2R106 knockdown inhibited quinine mediated adipogenesis. (**A**) The morphology changes of control and T2R106 knockdown cells were observed by microscopy on Day 14 of differentiation (magnification 100×); and (**B**) Expression levels of PPARγ, C/EBPα and FABP4 in shScramble and shT2R106 cells were detected by qRT-PCR. Cells were induced for differentiation with MDI for 14 days, total RNA was isolated and used for detection of PPARγ, C/EBPα and FABP4 mRNA expression levels with qRT-PCR (mean ± SEM; *n* = 6; two way ANOVA; * *p* < 0.05, ** *p* < 0.01, *** *p* < 0.001, indicated fold change compared with shScramble at the indicated time).

**Figure 7:**
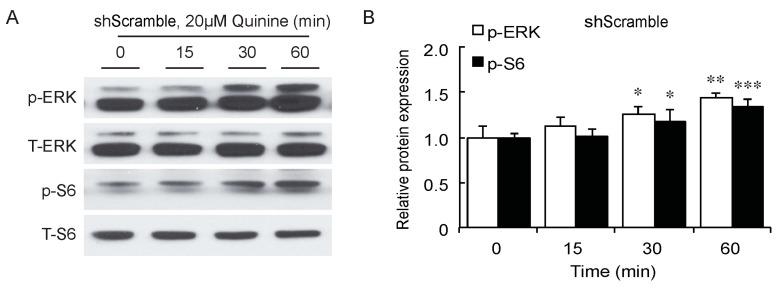

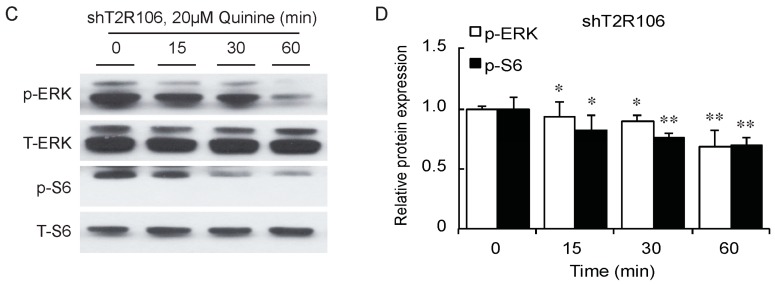
T2R106 knockdown impaired quinine mediated ERK/S6 signaling activation. (**A**) Expression of phospho-ERK (p-ERK) and phospho-S6 (p-S6) proteins in shScramble cells by Western blot; (**B**) Quantification of p-ERK and p-S6 protein expression; (**C**) Expression of p-ERK and p-S6 proteins in shT2R106 cells by Western blot; and (**D**) Quantification of p-ERK and p-S6 protein expression. Control and T2R106 knockdown mouse preadipocytes were serum starved for 2 h with HBSS, cells were then treated with 20 µM quinine for the indicated time. Cell lysates were then used for immunoblotting for ERK, p-ERK, S6 and p-S6 by Western blot (* *p* < 0.05, ** *p* < 0.01, *** *p* < 0.001, indicated fold change compared with 0 min).

As a bitter compound [8,9,10,11], one of the possibilities for quinine-mediated adipogenesis is to function through a bitter taste receptor. Previous reports showed that the bitter taste receptor T2R106 is expressed in mouse adipose tissue and its expression was significantly changed in high fat diet treated mice, suggesting that T2R106 might have a regulatory function in adipose tissue [20]. To determine whether bitter taste receptor T2R106 is the mediator for the pro-adipogenic effect of quinine, we measured quinine-induced adipogenesis in the absence of T2R106 and observed knockdown of T2R106 impaired quinine mediated adipogenesis. As shown in Figure 5A, expression of T2R106 increased during preadipocyte differentiation, which hints at its potential role in adipogenesis. To confirm this, knockdown of T2R106 was performed. T2R106 shRNA was effectively transduced into mouse primary preadipocytes (as indicated by GFP, Figure 5C), and T2R106 expression was significantly decreased with shRNA knockdown (Figure 5B). T2R106 knockdown and control cells were then induced for adipogenesis in the presence of quinine. The result showed that the accumulation of lipid droplets and expression of adipogenic genes in T2R106 knockdown cells were obviously decreased compared with control cells (Figure 6A,B). In addition, quinine was shown to increase the phosphorylation levels of ERK and S6 in control cells (Figure 7A,B). However, knockdown of T2R106 impaired the activation of ERK/S6 signaling mediated by quinine (Figure 7C,D). These results suggest that the pro-adipogenic effect of quinine functions through ERK/S6 pathways, and T2R106 at least partly influences quinine-mediated adipogenesis.

## 3. Discussion

Conflicting data exist in the literature on the regulation of quinine mediated body weight gain and adipogenesis. We report here quinine robustly induced adipogenesis of both mouse primary preadipocytes (Figure 1) and 3T3-L1 preadipocytes (Figure 3C), with increased lipid accumulation, adipogenic marker expression and glucose uptake (Figure 1). Our data are consistent with prior reports [19,20], in which long-term use of quinine resulted in excessive body weight gain and type 2 diabetes in humans [19], and addition of quinine has been shown to robustly stimulate 3T3-L1 adipogenesis [20]. However, very different findings were reported in rats and mice. Although quinine was reported to control body weight in both mice and rats [13,14,16,18], quinine decreased food intake in rats without affecting food intake in C57BL6/J mice. The basis for the difference in the *in vivo* research could be related to the duration and concentration of quinine supplementation, or the specific dietary content used for feeding rats and mice.

Although quinine was reported to affect food intake and body weight in *in vivo* animal models [13,15,16,17,18], seldom these groups tested its effects on adipocyte differentiation and metabolism in an *in vitro* cell model, which would add support to their observations in *in vivo* animal models. Until recently, quinine was reported to induce weight loss of HFD-induced obese mice, resulting in a decrease in fat pad weight and adipocyte size without affecting the weight of other organs and energy intake [21]. Meanwhile, 100 µM quinine supplementation inhibited 3T3-F442A preadipocyte differentiation by decreasing lipid accumulation and expression of adipogenic markers [21]. In contrast, our study shows for the first time that quinine robustly stimulated adipogenesis in both mouse primary preadipocytes (Figure 1 and Figure 2) and 3T3-L1 preadipocytes (Figure 3C,D). The reason for this difference could be that a different cell type and quinine concentration was used in a different context. What is interesting, however, is that 50 µM quinine presents an effective concentration for inducing adipogenesis in mouse primary preadipocytes based on our results, and over 100 µM quinine treatment caused an unhealthy morphology change in 3T3-L1 cells (Figure 3C,D), while 100 µM quinine shows an inhibitory effect on 3T3-F442A preadipocyte differentiation [21]. The disparities between our work and other groups can hopefully be resolved with further research.

Quinine, caffeine, 6-propyl-2-thiouracil (PROP), sucrose, salicin, hesperidin and sodium benzoate are substances well known for their bitter taste and function as bitter agonists [22]. These bitter compounds have been reported to be involved in the regulation of obesity and adiposity. Caffeine supplementation attenuates HFD-induced obesity in rats by decreasing levels of cholesterol, triglycerides, free fatty acid and the size of adipose tissue [23]. In addition, taste blindness to bitter tasting of PROP has been identified as a genetic marker for food choice and adiposity in human [24]. Furthermore, high sucrose diet results in hepatic steatosis, obesity and diabetes in mice, which are commonly associated with increased body weight gain and insulin tolerance [25,26]. A combination of glucosyl hesperidin (G-hesperidin) and caffeine resulted in decreased body weight, adipose tissue weight and liver lipogenesis in HFD-induced mice, in which lipogenesis markers like sterol regulatory element-binding protein-1c (SREBP-1c) and fatty acid synthase (FAS) were robustly decreased compared with that in control mice, but none of these were observed in mice fed each alone [27]. However, within the seven bitter compounds tested in this study, only supplementation of quinine showed pro-adipogenic effect in mouse 3T3-L1 preadipocytes, while the other bitter compounds had no effect on mouse adipogenesis (Figure 3). One possibility for this difference could be the concentrations of bitter agonists tested were not at an effective concentration. However, few effects of bitter agonists were tested and confirmed in the *in vitro* cell model, which makes it worthwhile but also challenging to test in further experiments.

In mammals, it is well accepted that the sense of taste includes bitter, sweet, umami, sour and salty [28]. Among them, bitter taste plays an essential role in host defense as a warning signal [29], which protects the animal against the ingestion of toxic components by direct taste aversion and nausea [28,30]. Bitter taste is sensed by bitter taste receptors (T2Rs), 25 T2Rs mediate bitter taste sensation in human and more than 30 T2Rs mediate bitter taste sensation in mouse [31,32,33,34]. Although the number of T2Rs varies among different species, most human T2Rs have similar homologs in mouse [33,34]. Individual T2Rs respond to diverse bitter tasting compounds, one bitter agonist may stimulate more than one T2R [35,36,37]. In our study, T2R106 expression increased during adipogenesis (Figure 5) and knockdown of T2R106 impaired quinine-mediated adipogenic effects instead of totally blocking quinine-mediated pro-adipogenesis (Figure 6), this suggest that quinine may activate more than one of the T2Rs.

The T2Rs not only exist in the gustatory system, but are also expressed in other tissues such as the respiratory system, gut system and adipose tissue [20,38]. In the gustatory and organ systems, bitter taste receptors mainly function to prevent ingestion of toxic substances [39,40,41]. In the respiratory tract, activation of T2Rs results in bronchodilation in response to inhalation of bitterants [39,41]. In the gut system, SREBP-2 stimulated T2Rs expression in cultured mouse enteroendocrine cells, and mT2R138 was shown to be a target of SREBP-2. During low cholesterol feeding, SREBP-2 enhanced mT2R138 expression to block dietary toxins, which further result in an increase in CCK secretion to slow gastric emptying and improve fat absorption in mice [40,42]. Expression of T2R106 in adipose tissue was significantly changed with altered fat tissue weight in HFD-induced mice, suggesting it could be involved in regulation of adipose tissue development [20]. Consistently, in this report, quinine supplementation activated ERK/S6 signaling which is known to be involved in adipogenesis [43,44], T2R106 knockdown impaired quinine mediated adipogenesis by inhibition of ERK/S6 signaling activation (Figure 7). These results suggest that the adipogenic effects of quinine are at least partly mediated by the modulation of ERK/S6 signaling and T2R106. ERK/S6 is one of the main signaling pathways involved in regulation of adipogenesis [43,44]. Whether ERK/S6 signaling is regulated by quinine or T2R106 through direct or indirect ways is still not clear. Moreover, further research is necessary to better characterize the mechanisms by which quinine mediates its effect on adipogenesis in the absence and presence of T2Rs.

Until now, expression of four T2Rs in human (h) and mouse (m) has been reported in the adipogenic system including hT2R46, mT2R106, mT2R108 and mT2R135, suggesting their effect on adipocyte development and metabolism in combination with bitter agonists [20,21,45]. Although further research is required to clear the mechanisms by which quinine functions on adipogenesis, the mild pro-adipogenic differentiation of quinine without induction components hints not only at specific adipogenic pathways but also that taste sense pathways are potential targets for further molecular mechanism research.

## 4. Materials and Methods

### 4.1. Reagents

DMEM (Dulbecco’s Modified Eagle Medium), FBS (fetal bovine serum), Collagenase Type I were purchased from Gibco (Life Technologies, Carlsbad, CA, USA). Restriction enzyme BglII and HindIII were bought from New England Biolabs (Ipswich, MA, USA). Lipofectamine 2000 was obtained from Invitrogen (Carlsbad, CA, USA). Antibodies for C/EBPα, PPARγ and FABP4 were purchased from Santa Cruz Biotechnology (Santa Cruz, CA, USA). Antibodies for HSL, p-HSL, ERK, p-ERK, S6 and p-S6 antibodies were purchased from Cell Signaling Technology, Inc. (Danvers, MA, USA). Methylisobutylxanthine, dexamethasone, Insulin, Oil Red O, Quinine hydrochloride dehydrate, Caffeine, Salicin, 6-propyl-2-thiouracil (PROP), Sucrose octaacetate, Hesperidin and Sodium Benzoate were bought from Sigma-Aldrich Co. (St. Louis, MO, USA). All tested bitter compounds were selected based on common knowledge and previous publications mentioned above. The bitter agonists were either dissolved in DPBS or DMSO, and final DMSO concentration did not exceed 0.1% (*v*/*v*) to avoid toxic effect on cells.

### 4.2. Cell Culture

Mouse primary preadipocytes were isolated from inguinal fat depot of 4-week-old C57BL/6 mice and grown in 10% calf serum/DMEM media. For differentiation, two days after confluency (Day 0), cells were stimulated with MDI/DMEM induction media (including 10% fetal bovine serum, 0.5 mM methylisobutylxanthine, 10 µg/mL insulin and 1 µM dexamethasone, MDI). After two days (Day 2), the media was changed to Insulin/DMEM media (containing 10% FBS and 10 µg/mL insulin, DI). Two days later (Day 4), cells were fed with 10% FBS/DMEM media until fully differentiation. Mouse 3T3-L1 cells were maintained and differentiated in the same way as primary cells.

### 4.3. Plasmids

T2R106 was stably knocked down by expression of an shRNA from the pSuperior.retro.neo vector (OligoEngine, Seattle, WA, USA). The shRNA was designed according to the manufacturer’s instructions to target the following sequence of T2R106: 5’-GGCAGGTTTACCTCTATAAGA-3′. Sequences for shRNAs used to generate the two other T2R106 knockdown cell lines (data not shown) are available upon request.

### 4.4. Oil Red O Staining and Quantification

Lipid accumulation of mouse primary preadipocytes was evaluated by Oil Red O stain. Briefly, after fully differentiation, wash cells with DPBS for 3 times, and then fixed with 10% formaldehyde for 1 h at room temperature. Rinse cells with 60% isopropanol for 10 min and incubate cells with Oil Red O working solution for 20 min. Cells were washed in H_2_O and photographed. Oil Red O was eluted by adding 100 µL of 100% isopropanol per well (in 24 well plates), and incubated for 10 min. 300 µL of 60% isopropanol was added to each well. 250 µL of each sample was transferred into a 96 well plate and read at 492 nm.

### 4.5. qRT-PCR

Total RNA was extracted from cells using TRIzol (Invitrogen) as described. Contaminating genomic DNA was removed from mRNA by using DNA-free^TM^ Kit (Life Technologies). Total RNA was reverse transcribed with RNA Reverse Transcription kit (Applied Biosystems, Foster City, CA, USA) according to the manufacturer’s protocols. Gene expression was measured by qPCR using MyiQTM real-time PCR detection system (Bio-Rad Laboratories, Hercules, CA, USA). Primers for T2R106: Forward: 5′-CACCAGCCTCAACCTCTTCT-3′, Reverse: 5′-GTGGGAAAGCAATTGACCAT-3′. Primers for PPARγ, C/EBPα, FABP4 and TBP were as described previously [46]. TBP was used as internal control.

### 4.6. Western Blot

Cell extracts were lysed with lysis buffer (0.5% Triton X-100, 2 mM EDTA, 150 mM NaCl, 1 mM PMSF, 50 mM Tris-HCl at pH 7.5) and quantified with BCA assay (Thermo Scientific, Waltham, MA, USA). After adding 4XSDS loading buffer and heating, protein samples were separated by Bis-Tris polyacrylamide gels (Life Technologies), transferred onto PVDF membranes (Millipore Corporation, Bedford, MA, USA) and immunoblotted with antibodies specific for PPARγ, C/EBPα, FABP4, ERK, p-ERK, HSL, p-HSL, S6 and p-S6.

### 4.7. Lipolysis

Lipolysis assays were performed in fully differentiated mouse primary adipocytes at Day 14 after induction of adipogenesis. Secretion of glycerol and NEFA (non-esterifed fatty acid) from cultured adipocytes into HBSS was determined with assay kits from Sigma-Aldrich Co. (FG0100), and Wako Diagnostics (Richmond, VA, USA; NEFA-HR2). Cells were treated with the indicated concentration of quinine or 10 µM forskolin for 2 h.

### 4.8. Glucose Uptake Assay

Fully differentiated mouse primary adipocytes were used for glucose uptake assay. Briefly, adipocytes were washed twice in PBS, and serum-starved in HBSS with 0.5% BSA for 4 h. After washing with Krebs–Ringer HEPES (KRH) buffer, the cells were incubated in KRH containing 4 nM insulin for 20 min to activate glucose transporter. After the insulin stimulation, 0.1 μCi/mL of 2-[^14^C] DOG (Perkin Elmer, Waltham, MA, USA) was added to incubate for another 10 min. Then, the cells were washed three times in ice-cold PBS, solubilized in 0.1% SDS. 2-DOG uptake was detected in liquid scintillation fluid using scintillation counter. For non-specific glucose uptake, cells were treated with 50 µM cytochalasin B to block translocation and get a background reading.

### 4.9. Statistical Analyses

All data are presented as mean ± SEM and were determined by Student’s *t*-test or ANOVA. The differences were indicated as follows: * *p* < 0.05, ** *p* < 0.01 and *** *p* < 0.001.

## 5. Conclusions

Taken together, our study demonstrates that quinine stimulates adipogenesis of mouse primary preadipocytes through the ERK/S6 pathway without affecting lipolysis. Among the tested bitter agonists, quinine is the only bitterant that showed a pro-adipogenic effect on mouse preadipocyte differentiation, suggesting its special role in adipogenesis. Knockdown of T2R106 impaired quinine-induced adipogenesis instead of totally blocking quinine’s adipogenic activity, suggesting a partial contribution of T2R106 to quinine dependent adipogenesis.
